# Shaping T Cell – B Cell Collaboration in the Response to Human Immunodeficiency Virus Type 1 Envelope Glycoprotein gp120 by Peptide Priming

**DOI:** 10.1371/journal.pone.0065748

**Published:** 2013-06-11

**Authors:** N. Kalaya Steede, Blake J. Rust, Mohammad M. Hossain, Lucy C. Freytag, James E. Robinson, Samuel J. Landry

**Affiliations:** 1 Department of Biochemistry and Molecular Biology, Tulane University School of Medicine, New Orleans, Louisiana, United States of America; 2 Department of Microbiology and Immunology, Tulane University School of Medicine, New Orleans, Louisiana, United States of America; 3 Department of Pediatrics, Tulane University School of Medicine, New Orleans, Louisiana, United States of America; National Institute of Allergy and Infectious Diseases, United States of America

## Abstract

Prime-boost vaccination regimes have shown promise for obtaining protective immunity to HIV. Poorly understood mechanisms of cellular immunity could be responsible for improved humoral responses. Although CD4+ T-cell help promotes B-cell development, the relationship of CD4+ T-cell specificity to antibody specificity has not been systematically investigated. Here, protein and peptide-specific immune responses to HIV-1 gp120 were characterized in groups of ten mucosally immunized BALB/c mice. Protein and peptide reactivity of serum antibody was tested for correlation with cytokine secretion by splenocytes restimulated with individual gp120 peptides. Antibody titer for gp120 correlated poorly with the peptide-stimulated T-cell response. In contrast, titers for conformational epitopes, measured as crossreactivity or CD4-blocking, correlated with average interleukin-2 and interleukin-5 production in response to gp120 peptides. Antibodies specific for conformational epitopes and individual gp120 peptides typically correlated with T-cell responses to several peptides. In order to modify the specificity of immune responses, animals were primed with a gp120 peptide prior to immunization with protein. Priming induced distinct peptide-specific correlations of antibodies and T-cells. The majority of correlated antibodies were specific for the primed peptides or other peptides nearby in the gp120 sequence. These studies suggest that the dominant B-cell subsets recruit the dominant T-cell subsets and that T-B collaborations can be shaped by epitope-specific priming.

## Introduction

Gradually emerging correlates of protection against human immunodeficiency virus type 1 (HIV-1) and simian immunodeficiency virus (SIV) include antibody against the envelope glycoprotein, antibody avidity, and CD4+ T-cell responses [1], [2], [3]. The role of CD4+ T-cell responses may be particularly important for affinity maturation of the B-cell response because studies on the ontogeny of broadly neutralizing antibodies have revealed an exceptionally large amount of divergence from germline antibody genes [4], [5]. Promising immunization regimens involve a prime and boost that differ in route and/or substance of the immunogen [1], [2], [3]. The partial success of these regimens could be due in part to improved collaboration of antigen-specific T cells and B cells. Understanding how immune priming influences B-cell development and specificity could lead to immunization strategies that focus antibodies onto epitopes that are associated with protection, such as the CD4 binding site and V2 loop [6].

Effective antibody production depends on CD4+ helper T-cell function, which is stimulated by antigen presentation and CD40 on the B cells and mediated by cytokine signals from the responding T cells [7], [8], [9], [10]. Although B-cells process antigen and present peptides to T cells, the spectrum of antigen-specific T cells is initially determined by antigen presentation from dendritic cells (DCs) at an earlier stage of the immune response. The profile of T cells typically reveals dominance of certain epitopes, which is determined by the availability of antigen sequences arising from antigen processing [11], [12], [13], peptide affinity for the class II major histocompatibility molecules [14], and the frequency of naïve T cells emerging from the thymus [15].

In the lymph nodes, interactions between antigen-specific B cells and T-follicular helper cells (Tfh) support the development of germinal centers (GC). The differentiation of primed T cells into Tfh depends on contact with both DCs and B cells that present the cognate epitope [16]. Tfh cells are characterized by distinctive cell-surface proteins that are responsible for retaining Tfh cells in the lymph node and maintaining the GC structure [17]. Some of the B cells differentiate into circulating plasma cells that secrete immunoglobulins, while others remain in the GC and undergo affinity maturation under the continued influence of Tfh cells.

In spite of well-established dependence of B-cell development on T-cell help, there are surprisingly few reports of any correlation between intensity of antibody and intensity of T cell responses [18], [19], [20]. Moreover, it remains unclear whether any particular specificity of T-cell is more important than another for helping a particular specificity of B-cell. T-B collaboration or T-B reciprocity refers to the phenomenon in which a particular specificity of B cell presents a T-cell epitope more efficiently than B cells with other specificities [21]. As a result, the collaborating B cells receive more help than other B cells from the collaborating T cells. The B-cell antigen receptor can affect T-cell epitope presentation by inhibiting or favoring certain pathways of antigen processing [22]. In spite of the evidence supporting a mechanism for T-B collaboration in vitro, little evidence has emerged from experiments in vivo. In small pox vaccinees, antigen-specific T-cell help was shown to correlate with antibodies against the same antigen [19]. However, relationships of epitope-specific T-cell and B-cell responses within a single antigen have not been reported.

In the present study, we sought to identify correlations between antibody and T-helper responses following immunization of mice with HIV_89.6_ gp120dss378. The gp120dss378 lacks the 378–441 disulfide bond and was previously found to induce a higher level of antibodies that prevent binding of CD4 to gp120 (CD4-blocking antibodies) in immunized mice [13]. While the gp120-specific antibody titer was poorly correlated with the bulk T-cell response, crossreacting and CD4-blocking antibody titers correlated well with the bulk T-cell response, as reported by IL-2 and IL-5 secreted from splenocytes restimulated with gp120 peptides. Numerous epitope-specific T-B correlations suggested that dominant B cells attract help from multiple T-cell lines. When the animals were primed with a gp120 peptide, antibody no longer correlated with the bulk T-cell response, but epitope-specific T-B correlations were still observed, most frequently involving antibodies against the primed peptide and peptides nearby in the gp120 sequence. The results suggest that epitope-specific immune priming fosters collaboration of particular B cells with the dominant T cells.

## Materials and Methods

### Proteins

The construction of baculoviruses expressing wild-type HIV_89.6_ gp120 and variant gp120dss378 was described [13]. For construction of the baculovirus expressing ODC-B, the pFastBac-1 derivative encoding gp120 was submitted to rounds of site-directed mutagenesis in order to delete sequences encoding the following protein segments: 29–255, 300–327, 396–405, 418–432, and 480–508. The three internal segments were each replaced with a codon for glycine. High Five cells (Invitrogen) were infected for 72 hours with passage-3 high-titer virus at a dilution that produces synchronous infection. Culture supernatants were stored at −76°C until use. Proteins were purified by affinity chromatography using a *Galanthus nivalis* lectin (GNL) column (Vector Laboratories) and a HisTrap column (GE Healthcare). The purified protein was stored in phosphate buffered saline (PBS) with 10% glycerol at −76°C until needed. Upon thawing, the concentration of protein in the sample was assessed using the BCA assay (Thermo Scientific) as per the manufacturer’s instructions. For expression of gp120_96ZM651.8_ in mammalian cells, the gene was amplified from p96ZM651gp120-opt, provided through the NIH AIDS Research and Reference Reagent Program from Drs. Yingying Li, Feng Gao, and Beatrice H. Hann [23]. Primer design incorporated restriction enzyme cleavage sites and encoded a C-terminal hexahistidine fusion. The amplification product was cleaved with restriction enzymes BamHI and XhoI and ligated into pcDNA3.1+Zeo:IntA [24] to create pKS700. For transfection of 293T cells, 6 µg plasmid DNA was diluted in Dulbecco’s Modified Eagle Medium (DMEM) to a final volume of 100 µL. In a separate tube, 21 µL FuGene HD (Roche) was diluted with 750 µL of DMEM. The diluted DNA and FuGene were combined with gentle mixing and then incubated at room temperature for 30–45 minutes. Medium was removed from a confluent cell culture in a T75 flask, and the transfection mix was applied drop-wise across the surface of the cells, and then the flask was gently rocked to distribute the transfection mixture. The culture was incubated at 37°C with 5% CO_2_ for 6 hours; the medium was exchanged with 20 µL 293II SFM (Invitrogen); and the incubation was continued for 3 days. Cell debris was removed by centrifugation for 10 min at 2500×*g*, and the gp120 was purified as described above.

### Peptides

Peptide 2 (GVPVWREATTTLFCASDAKA), peptide 29 (SIGPGRAFYARRNIIGDIRQ), and the control peptide (TPAETTVRLRAYMNTPGLPV) were synthesized by JPT Technologies. The 38 peptides (20-mers overlapping by 10 residues) spanning residues 40–109 and 180–508 of gp120 sequence were synthesized and distributed into microtiter wells by JPT Peptide Technologies. Peptide sequences (Table S1) including cysteines as the carbamidomethyl derivatives were as described [25]. For the T-cell restimulation assays, 0.4 µg of each peptide was dissolved in 30 µL complete medium. For ELISA, peptides were dissolved at a concentration of 4 µg/mL in freshly prepared 0.1 M NaHCO_3_, pH 8.1.

### Immunization

Six- to eight-week old female BALB/c mice from Charles River Laboratory were used for this study. Starting one week after arrival at the animal facility, mice received a series of three intraperitoneal injections (days -9, -6, and -3) of 100 µL peptide emulsion in incomplete Freund’s adjuvant (IFA), PBS-IFA emulsion, or PBS. Peptide-primed groups received 100 µg peptide 2, peptide 29, or control peptide that had been dissolved in PBS and then emulsified with IFA. Starting on day 0, some mice also received three biweekly intranasal administrations of 20 µg gp120dss378 plus 5 µg mutant (R192G) heat-labile toxin [26] as adjuvant (kindly provided by Dr. John Clements) in a total volume of 10 µL. The mice were sacrificed one week after the last boost. Cardiac blood and spleen were collected from each mouse immediately after sacrifice. This study was carried out in strict accordance with the recommendations in the Guide for the Care and Use of Laboratory Animals of the National Institutes of Health. The protocol was approved by the Institutional Care and Use Committee of Tulane University School of Medicine (Protocol Number: 4103R).

### Analysis of Serum IgG and IgM Antibodies by ELISA

For measurement of antigen-specific serum IgG levels, wells of 96-well EIA/RIA plates (Costar) were coated by incubation for 1 hour with 100 µL antigen solution containing 2.5 µg/ml protein in PBS or 4 µg/ml peptide in 100 mM sodium carbonate, pH 8.1. After washing 5 times, plates were blocked with PBS containing 0.5% (v/v) Tween-20, 4% (w/v) whey and 10% (v/v) fetal bovine serum (blocking buffer) in a final volume of 200 µL for 30 minutes. After the blocking buffer was removed, wells were incubated for 1 hour with serum diluted in blocking buffer, followed by washing 5 times. The wells were incubated with goat anti-mouse IgG conjugated with horseradish peroxidase (HRP) (Invitrogen, M30107) or goat anti-mouse IgM conjugated to alkaline phosphatase (AP) (Sigma, A7784) at 1∶2000 dilution in blocking buffer for 1 hour. HRP was detected by [0.02% 3,3′,5,5′-tetramethylbenzidine (TMB) and 0.01% hydrogen peroxide in 0.1 M sodium acetate buffer, pH6.0] and allowing the color to develop for 3 minutes. The reaction was stopped with 1 M phosphoric acid, and absorbances were read at 450 nm. AP was detected by 4-nitrophenyl phosphate disodium salt hexahydrate (Sigma, N9389) and allowing the color to develop for 20 minutes. The reaction was stopped with 2 N sodium hydroxide. Values of A_450_ were fit by non-linear regression in Prism (GraphPad) to a four-parameter curve with variable slope, and the value of EC_50_ was taken as the antibody titer. For analysis of CD4 blocking, binding of soluble CD4 (sCD4) to gp120_89.6_ after reaction with diluted serum was detected with biotinylated guinea pig anti-CD4 (prepared in-house) and HRP-streptavidin. Values of A_450_ normalized to percent-bound sCD4 were fit by non-linear regression to a curve with variable slope, and the value of EC_50_ was taken as the antibody titer.

### T-cell Restimulation

Splenocytes were isolated as previously described [25], except that spleens were dissociated using a GentleMACS dissociator (Miltenyi Biotec), following the manufacturer’s protocol. The tissue was transferred to a 40 µm cell strainer (Falcon BD) followed by a 5 mL wash with PBS, pH 7.2, 0.5% bovine serum albumin (BSA), and 2 mM EDTA. The cells were collected by centrifugation (300×g) and resuspended in1 mL RBC Lysing Buffer (Sigma), incubated at room temperature for 2.5 min, and then diluted with 20 mL RPMI. The splenocytes were recovered by centrifugation (300×g), and cells were resuspended with 5 mL of complete medium (RPMI, 10% fetal bovine serum, 100 U/mL penicillin, 100 mg/mL streptomycin, and 2 mM L-glutamine). Cells were plated at a density of 4×10^5^ cells in 170 µL complete medium, with each well including a single peptide at 2.4 µg/mL. Each culture plate contained a single analysis for each of two mice. On day 3 of culture, 115 µL was removed for assay of cytokine secretion and 1 µCi of ^3^H-thymidine in 115 µL complete medium was added. After 18 hours, proliferation was assayed by harvesting the cells onto glass filters and scintillation counting. The counts per min for each condition was divided by the average counts per min for unstimulated cultures to obtain the stimulation index (SI). Cytokine concentrations in the culture supernatants were assayed using custom 9-plex Milliplex MAP kits (Millipore) according to the manufacturer’s protocol. The arrangement of supernatants on bead plates was a replicate of the culture plate, including two mice per plate. Bead plates were read on a Bio-Plex 200 (Bio-Rad), and data were processed with optimization of the standard curves in Bio-Plex Manager 6.0 (Bio-Rad). Inspection of results indicated that the standard curve was affected by the matrix and was subject to plate-to-plate variation. For IL-2, the lowest-value cytokine concentrations were an average of 9 pg/mL below baseline. In order to remove this source of variation, all experimental values of cytokine concentration for each mouse were adjusted to position the lowest value at 1 pg/mL.

### IL-2 ELISpot

All steps prior to development were performed in sterile conditions. Day 1, wells of a 96 well Unifilter 350 Polyfiltronics plate (Whatman #7770–0008) were pre-wet for 1 min with 15 µL 35% ethanol and washed 3 times with Dulbecco’s phosphate buffered saline (DPBS) (Invitrogen #14190–144). Wells were coated with 100 µL of anti-mouse IL-2 (eBioscience #14–7022–85) capture antibody at 0.01 mg/ml in DPBS. The plate was wrapped in Parafilm and incubated overnight at 4°C. Day 2, wells were washed with sterile wash buffer (DPBS +0.05% Tween20), blocked with 175 µL RPMI (Invitrogen 11875093) +10% FBS (Invitrogen #16140071); and the liquid was decanted. Splenocytes were added at 2.94×10^6^ cells/mL in 170 µL. A single peptide was added in a volume of 30 µL to achieve 0.4 µg/mL peptide. The plate was incubated at 37°C, 5% CO2 for 36 hrs, and then developed as follows. Wells were washed 5 times with wash buffer and once with distilled water, and then 100 µL anti-mouse IL-2 Biotin (eBioscience #13-7021-81) was added at 0.01 mg/ml in assay diluent (DPBS +10% FBS). The plate was incubated at room temperature for 3 hrs; wells were washed 6 times with wash buffer; and then 100 µL avidin-HRP (eBioscience 18-4100-94) was added at 1/1000 in assay diluent. The plate was incubated at room temperature for 45 min; wells were washed 4 times with wash buffer and 2 times with DPBS. For preparation of substrate solution, 4 mg of 3-amino-9-ethylcarbazole (AEC) (Sigma # A5754) was dissolved in 1 mL dimethylformamide, and diluted to 15 mL with 0.1-M phosphate-citrate, pH 5.0; the solution was passed through a 0.45 mm filter; and 10 mL 30% H2O2 (Sigma #H1009) was added just before use. Substrate solution (100 µL) was added to each well, and the plate was incubated at room temperature for 40 minutes. The plates were read in a CTL ImmunoSpot Analyser and analyzed with ImmunoSpot™ software.

### Statistical Analysis

T-cell and antibody responses were compared for different mouse groups using one-way ANOVA in Prism (GraphPad). For comparison of bulk T-cell responses across all groups, Tukey’s post test for multiple comparisons (GraphPad Prism) was used. For comparison of peptide-specific T-cell responses between gp120 peptide-primed and control-primed groups, Dunnett’s post test was used. Pearson correlations were evaluated in Prism.

## Results

**Figure 1:**
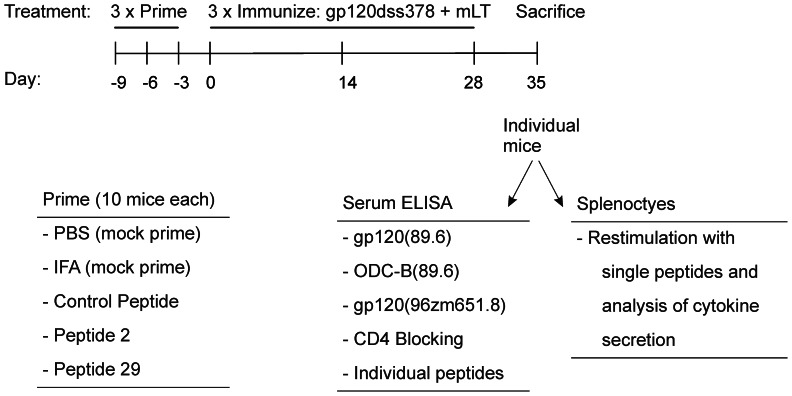
Immunization schedule, treatment groups, and analyses of immune response.

Since B cell development and affinity maturation depend on T-cell help, it was reasonable to expect a correlation between antibody levels and the average T-cell response (across all peptides), although there are very few reports of such correlations *in vivo* [18], [19]. In order to directly test for such a correlation, groups of 10 BALB/c mice were intranasally immunized with a disulfide-deletion variant of HIV_89.6_ gp120 (gp120dss378) that was previously found to raise more CD4-blocking antibodies than raised by the wild-type gp120 [13]. Two of the mouse groups were primed intraperitoneally with a single gp120 peptide in order to test the effect of peptide priming on protein immunization (Fig. 1). Peptide 2 lies near the N-terminus of gp120, and peptide 29 lies in the V3 loop. In previous studies, both peptides restimulated proliferation of splenocytes from BALB/c mice that had been immunized intranasally with gp120 or gp120dss378 [13], [25]. Peptide 29 also reacted with antibodies [13]. The control peptide from hepatitis C virus NS3 has no significant identity to gp120 or any protein that is predicted to be expressed in mice. Additional control groups of mice received IFA or phosphate-buffered saline (PBS) prior to the gp120dss378. One week after the last administration, the animals were sacrificed, and sera and spleens were analyzed for gp120-specific immune responses. All animals became immunized to the protein, as indicated by the development of antibody against the protein. Likewise, all primed animals developed significant antibody against the primed peptides.

T-cell responses were assessed by restimulation of splenocytes with individual peptides from an array of thirty-eight 20-mers (peptides 2–7 and 16–47), spanning all but variable regions V1–V2 of gp120, followed by analysis of the cytokines released into the culture supernatant. The logarithm of the cytokine concentration (pg/mL) was found more reliable than the actual cytokine concentration for the comparison of T-cell responses between mouse groups. For example, the largest peptide-specific differences in average log[cytokine] between mouse groups were also the most significant (lowest p-value); whereas, this was often not the case when using the untransformed cytokine concentration. We attribute the reliability of log[cytokine] to the fact that it emphasizes the low-range of measurable concentration. All peptide-specific T-cell and B-cell responses are available in Table S2 (Excel file).

### Correlation of Bulk T-cell and B-cell Responses

**Figure 2:**
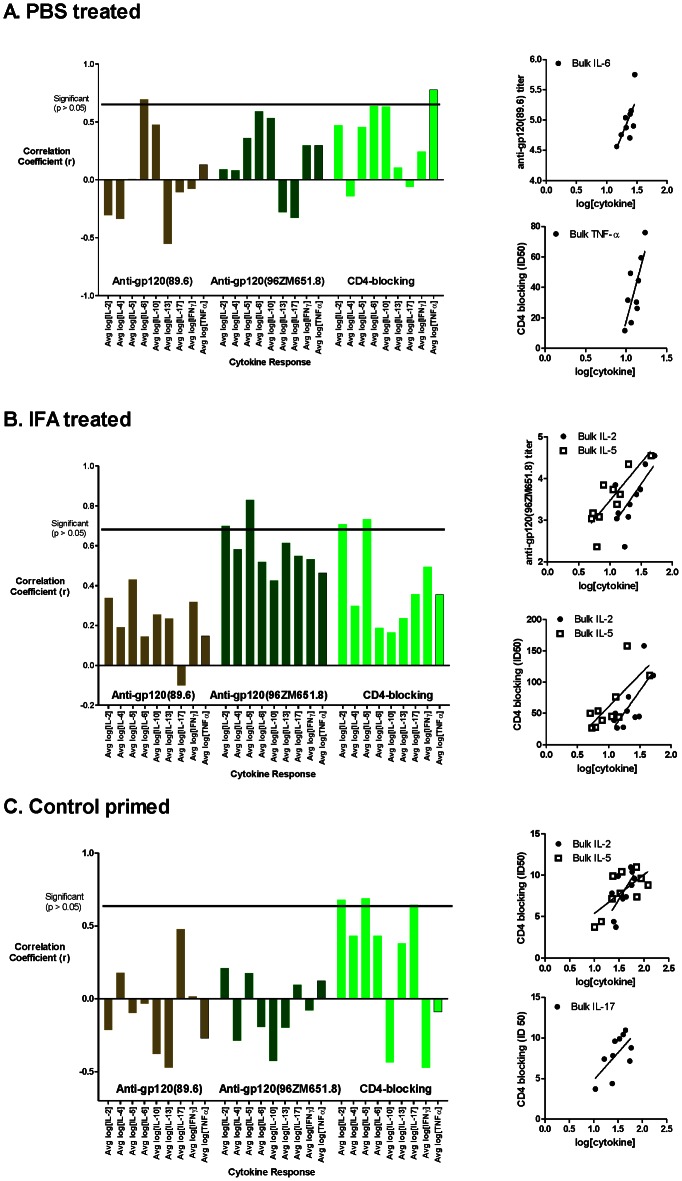
Correlation of antibody and bulk T-cell responses in the unprimed (A, B) and control-primed (C) mouse groups. Bar graphs indicate Pearson correlation coefficients for the bulk cytokine response with reactivity to the indicated antigens. Scatter plots illustrate the significant correlations (p<0.05). Bulk IL-6 and TNF-α correlated with gp120 and CD4-blocking antibodies, respectively, in the PBS-treated mouse group (A). Bulk IL-2 and IL-5 correlated with gp120_96ZM651.8_-crossreactive and CD4-blocking antibodies in the IFA-treated mouse group (B). Bulk IL-2, IL-5, and IL-17 correlated with CD4-blocking antibodies in the control-primed group (C).

The bulk cytokine secretion by peptide-stimulated splenocytes (average log[cytokine] secreted in response to the 38 peptides) was tested for correlation with the antibody titers for reaction with gp120, crossreaction with gp120 from HIV_96ZM651.8_ (gp120_96ZM651.8_), and competition with CD4 binding (CD4 blocking). Correlations for the bulk T-cell responses were only observed in the unprimed or control-primed groups. In the PBS-treated group, bulk IL-6 correlated with titer for gp120, and bulk TNF-α correlated with CD4 blocking (Fig. 2A). In the IFA-treated animal group, bulk IL-2 and IL-5 responses correlated with the antibody titer for crossreaction with gp120_96ZM651.8_ and ID_50_ for CD4 blocking, but not with the titer for reaction with gp120 (Fig. 2B). In the control-primed group, bulk IL-2, IL-5, and IL-17 correlated with ID_50_ for CD4 blocking (Fig. 2C). The lack of correlation in the peptide-2 and peptide-29 groups may be due to perturbations in the responses caused by the peptide priming, which were analyzed further below.

### Correlation of Epitope-specific T-cell and B-cell Responses

**Figure 3:**
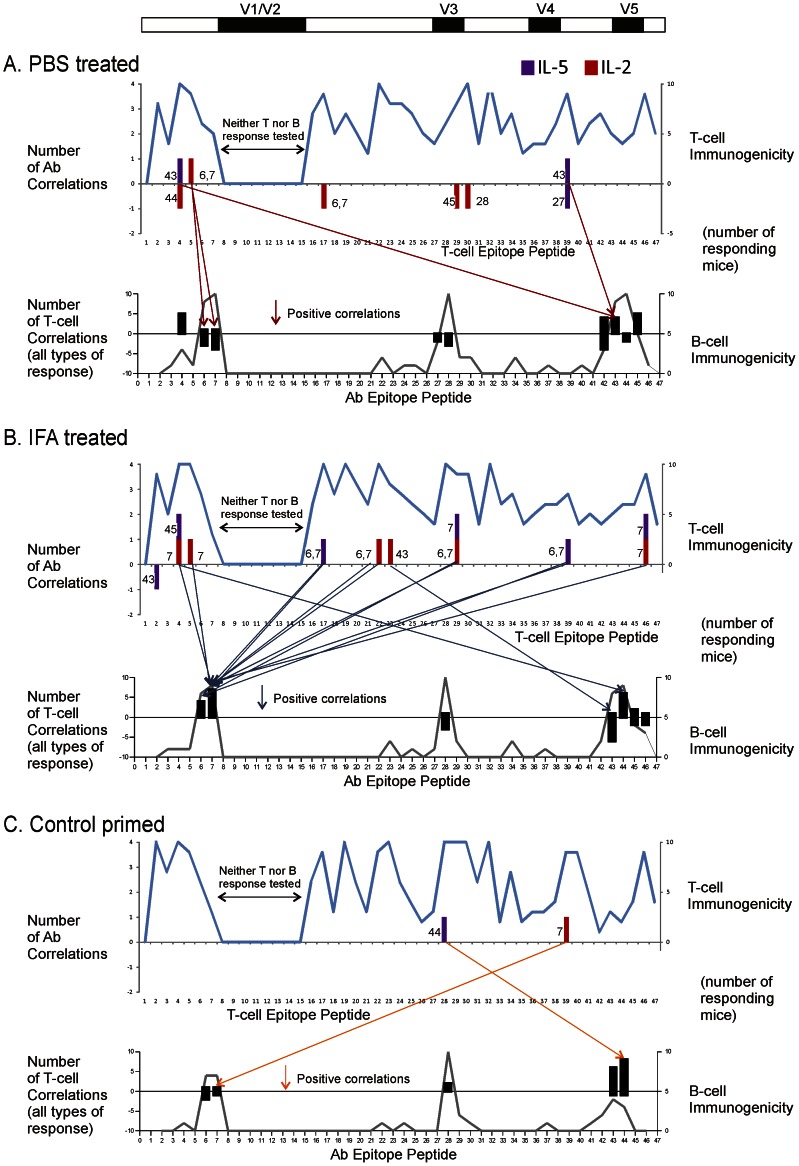
Epitope-specific T-B correlations in unprimed and control-primed mouse groups. The groups of mice are the same as in Fig. 2. Arrows connect positively correlated (*r* ^2^>0.42 and *p*<0.05) T-cell responses in the upper graph to B-cell responses in the lower graph. A unit in the *y*-axis indicates that at least one T-B correlation involved the indicated cell specificity. The T-cell specificities of only the correlations involving IL-2 and IL-5 are illustrated. The cytokine secreted in the T-cell response is indicated by the color of the bar, and the correlated B-cell specificity is indicated by the number next to the bar. [In a few cases, a single T-cell specificity and cytokine correlated with two B-cell specificities.] The B-cell specificities of all correlations (involving 9 cytokines or proliferation) are illustrated. Negative values indicate negative correlations. The line plots indicate epitope dominance as the number of responding mice. A positive T-cell response was identified by log[IL-2] greater than 2×SD for unstimulated wells, and a positive B-cell response was identified by A450 greater than 0.2. The analysis of correlation was limited to dominant T-cell and B-cell responses (see text). Positive correlations were more frequent than negative correlations. Many T-B pairings were observed in multiple mouse groups, e.g., T5–B7 in PBS-treated and IFA-treated groups and T39-B7 in IFA-treated and control-primed groups. Additional examples are presented in Table 1.

**Figure 4:**
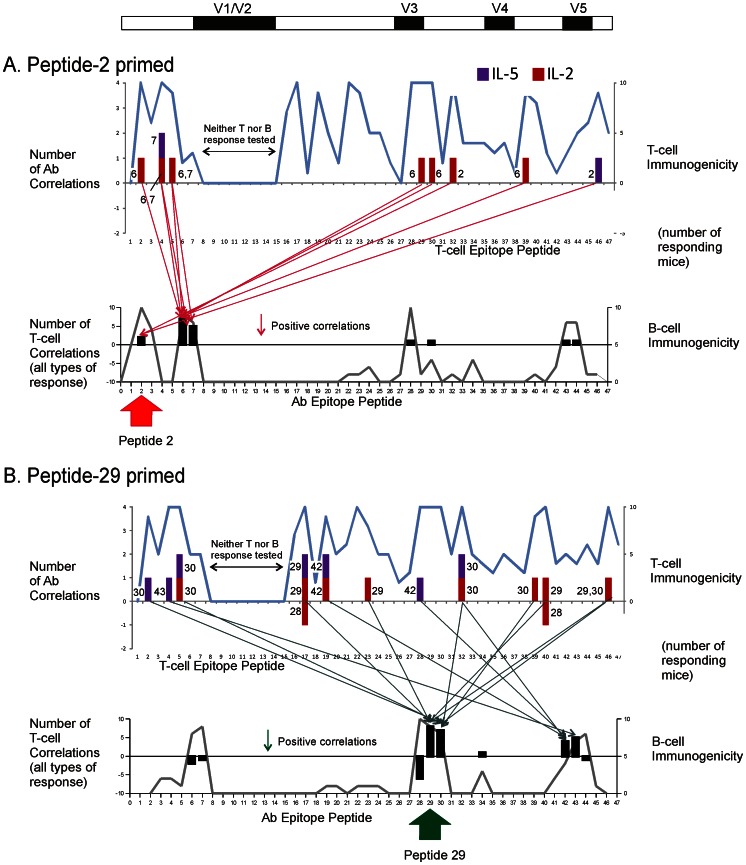
Epitope-specific T-B correlations in mouse groups primed with gp120 peptide 2 (A) and peptide 29 (B). The notation is as described in the legend for Fig. 3. The priming peptide is indicated below the graph of B-cell specificity. T-B correlations were concentrated on B-cell specificities near the priming peptide.

Numerous T-B correlations were identified for individual T-cell epitopes, involving antibodies for both linear and conformational epitopes (Figs. 3 and 4, Table 1). For the purposes of this study, an “individual” T-cell epitope is defined by the stimulatory activity of a single 20-mer peptide. Several criteria were employed in order to identify significant T-B correlations. The T-cell responses were limited to the set of 14 dominant-epitope peptides, which elicited significantly greater splenocyte proliferation than observed for unstimulated cells (Wilcoxon signed rank test). For the purposes of correlation, the peptide-specific antibody responses were limited to the set of peptides that reacted with serum (A_450_>0.2) in at least three mice of the group, which ranged from 5 to 10 peptides in the different mouse groups. Thus, as many as 14×10 or 140 T-B correlations were possible for a given cytokine, and nine cytokines were analyzed. Correlations were initially identified by values of *r* ^2^ greater than 0.42 and then were confirmed as significant (p<0.05). The number of correlated T-B pairs represented a small fraction (<10%) of the total possible in each mouse group.

**Table 1:** Response associated with T-B correlationsa.

		Peptide specificity of correlated T-cell response													
**Peptide specificity of correlated B-cell response**	***PBS***	2	4	5	17	19	22	23	28	29	30	32	39	40	46
	**4**		Prolif^b^		IL17,Prolif						IL17,IFNγ,TNFα	TNFα	IL6^c^		
	**6**			**IL2,IL6**	**(IL2)**				(IL13)	(IL17)					
	**7**			**IL2**	**(IL2)**				(IL13)	(IL17)			(IL13)		
	**27**											(IL13)	(IL5)		
	**28**		**(TNFα)**				(IL13)				(IL2),(TNFα)				
	**42**			IL17		**(IL6)**	**IL17**			**(IL2),IL6**	**IL6**		**(IFNγ)**		**(IL6)**
	**43**		**IL5**			**IFNγ**						**IFNγ**	**IL5**		
	**44**		(IL2),(IL6),(TNFα)		**(Prolif)**										
	**45**		**IL6,Prolif**		IL13,Prolif		IL13				IL17,TNFα	IL6,TNFα			
	**CD4** d			IL4,IL6			IL4,IL10,IL13,TNFα		(IL6)	(IL4)	IL2,IL4,IL10,IFNγ,TNFα	TNFα	IL2		
	**XR** e			IL6					(IL6)	(IL17)	IL4,IL10	TNFα			
	***IFA***	2	4	5	17	19	22	23	28	29	30	32	39	40	46
	**6**				**IL5**		IL2			IL2,IL17			IL5		
	**7**		**IL2**	**IL2**	**IL5**		IL2,IL4,IFNγ			IL2,IL5			IL4,IL5		IL2,IL5,IL17
	**28**		**(Prolif)**	**(Prolif)**	**(Prolif)**							**IL10,IFNγ**			
	**43**	**(IL5),(IL6),(IL10),(IL13),(IFNγ)**		**(IL4),(IL6),(IL10),(IL13),(IFNγ)**		**(IL6),(IL10),(IFNγ)**		**IL2**		**(IL10)**	**(IL10),(IFNγ)**	**(IL6),(IL13)**			
	**44**		**IL10,IFNγ**	**IFNγ**	**IL4,IL6,IL10,IFNγ,Prolif**		IL4							IL10,IL17	IFNγ
	**45**		**IL2,IL5,IL6**	IL2		(IL10)			(IL6), (IL17)						
	**46**				(IL13)		(IL6),(IL10),Prolif								
	**CD4**		IL5,IL10,IL13	IL2,IL17		IL2,IL5,IL17	IL2	(IL4)	(IL6)	IL2,IL5	IL4,IL5	IL5			IL5
	**XR**			IL2,IL5	IL5	IL5,IL17	IL2,IL4,L5,IL13			IL2,IL4,IL5,IL6	IL5	IL5			IL2,IL4,IL5,IL6,IL17,TNFα
	***Control***	2	4	5	17	19	22	23	28	29	30	32	39	40	46
	**6**				**(IL13)**				(IL6)				IL4,IL6		
	**7**								(IL10)				IL2,IL6		
	**28**				**IL17,IFNγ**						TNFα				
	**43**	**IL6,IL13**	**IL10**	**TNFα**				IL10	(IL17)		IL10				IL10
	**44**	**(IL10)**	IFNγ,Prolif	**Prolif**	**Prolif**	IL13,Prolif	IL13,IFNγ,Prolif	IL4,TNFα	IL5,IL13			IL13,Prolif			
	**CD4**	IL17	IL6,IL17	IL5		IL5		IL17			IL2,IL5, IL6,IL17		IL2,IL5		IL2,IL5,IL6
	**XR**			(IFNγ)											
	***Pep2***	2	4	5	17	19	22	23	28	29	30	32	39	40	46
	**2**											IL2			IL4,IL5,IL17
	**6**	IL2	IL2,IL10	**IL2**		IL6,IL10				IL2,IL4,IL6	IL2		IL2		
	**7**		**IL2,IL4,IL5,IL10**	**IL2,IL4,IL6**		IL6,IL10				IL4,IL6				IL6	
	**28**					IFNγ									
	**30**							**(IL17),(IFNγ)**							
	**43**			TNFα											
	**44**	IL4													
	**CD4**	(IL13),(TNFα)													
	**XR**													IL17	
	***Pep 29***	2	4	5	17	19	22	23	28	29	30	32	39	40	46
	**6**			**(IL4),(IL6)**									(IL4)		
	**7**			**(IL4),(IL6)**											
	**28**	(IL10)		**(IL17)**	**(IL2),(IL4),(IL6),(IL13)**						(IFNγ)	**(IL4)**		(IL2),(IL17)	
	**29**		Prolif		IL2,IL5, IL6,IL10, IL13, IL17,IFNγ	IL10		IL2				IL17	IL10	IL2	IL2,Prolif
	**30**	IL5,IL17	IL4	IL2,IL4,IL5,IL6,IL10,IL13					IL10			IL2,IL5	IL2		IL2,Prolif
	**34**							IL17							
	**42**					**IL2,IL4,IL5,IL6,IL13**			**IL4,IL5**	**(IL10)**		**IL6**			
	**43**		**IL5,IL10,IL13,TNFα**				IL10	IL10	Prolif				IL4		
	**44**										(IL17)				
	**CD4**								(IL4)	IL2					
	**XR**								IL4					(IL10)	

a Negative correlations indicated in parentheses.

b Proliferation.

c Bold underline indicates positive or negative correlation of same T and B cell specificity (not necessarily the same type of response) in multiple animal groups.

d Antibodies that block CD4 binding to gp120.

e Antibodies that crossreact with gp120_96ZM651.8._

**Figure 5:**
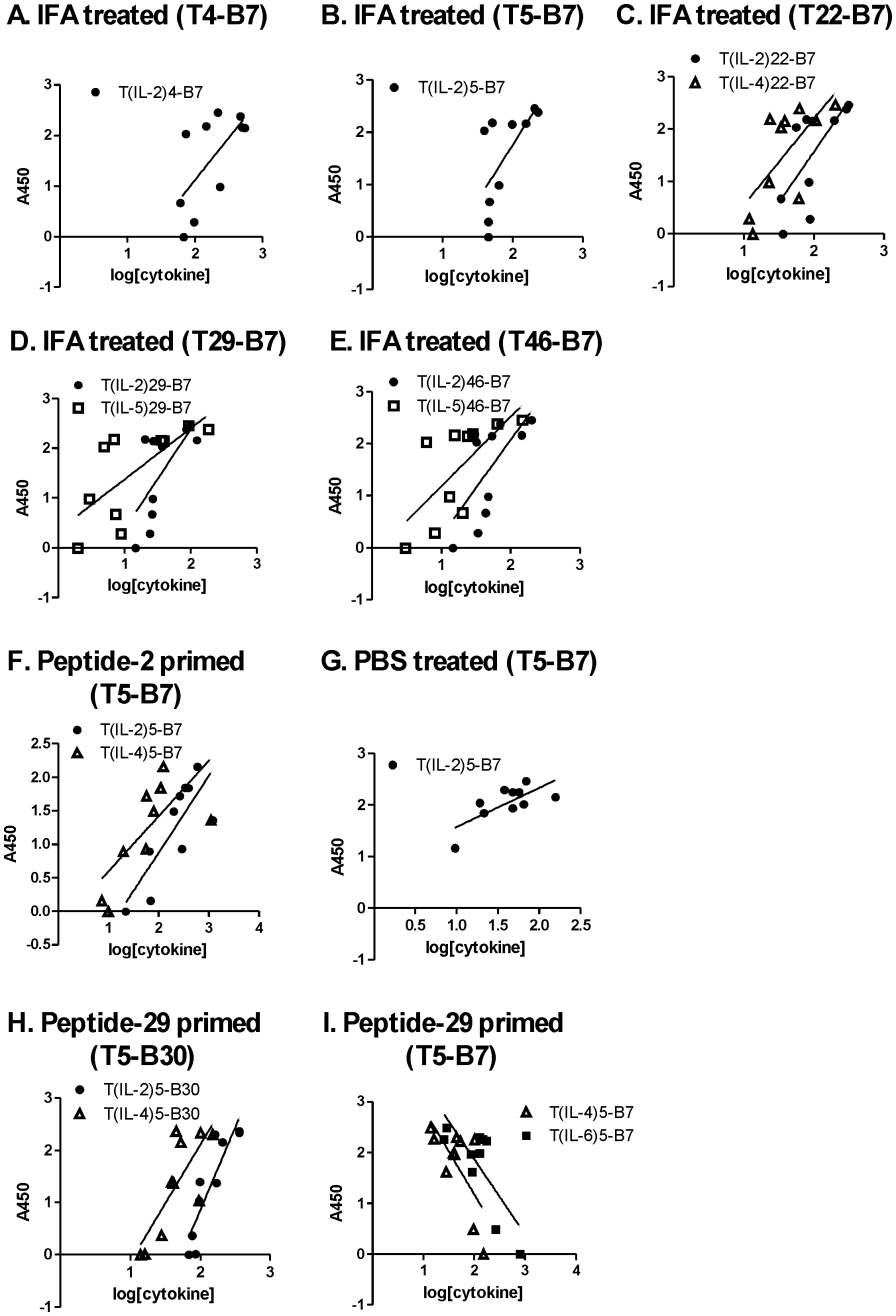
Scatter plots illustrate selected T-B correlations. The groups of 10 mice are the same as in Figs. 3 and 4. A-E. In the IFA-treated mouse group, peptide-7 antibodies correlated with log-concentration of IL-2, IL-4, and IL-5 secreted in response to the indicated peptides. F, G. In two additional mouse groups, peptide-7 antibodies correlated with the IL-2 and IL-4 response to peptide 5. H. Priming with peptide 29 induced correlation of peptide-30 antibodies with IL-2 and IL-4 responses to peptide 5. I. In the peptide-29 group, correlations of peptide-7 antibodies with peptide-5 T-cell responses became negative. The peptide specificities of the correlated T-cell and B-cell responses are indicated in the graph titles, and the associated cytokines are indicated in the legends for individual graphs.

Peptide-specific antibodies were frequently correlated with T-cell responses to multiple peptides. For example, in the IFA-treated mice, antibodies reacting with peptide 7 (response B7) correlated with five different IL-2 responses spread over the entire gp120 (responses T_IL-2_4, T_IL-2_5, T_IL-2_22, T_IL-2_29, and T_IL-2_46) (Fig. 3B, scatter plots in Fig. 5A–E). Some of the same T-B7 correlations were observed with T-cell responses identified by other cytokines (T_IL-4_22, T_IL-5_29, and T_IL-5_46) (Fig. 5C–E). It is possible that correlations involving multiple cytokines result from polyfunctional T cells, but this cannot be resolved with the present data.

A subset of correlations was observed in multiple animal groups. For example, T_IL-2_5-B7 was observed in the IFA, peptide-2, and PBS groups (Fig. 5B, 5F, 5G). If the type of cytokine and sign of correlation was disregarded, a majority of the correlated T-B pairs was observed in two or more animal groups (bold, underlined responses in Table 1). The two mouse groups sharing the largest number of T-B pairings in a positive correlation (seven) were the control and IFA groups.

In the control-primed mouse group, a single T-cell response (T_IL-17_40) was found to correlate with control-specific antibodies (data not shown). In the IFA-treated group, the same IL-17 response correlated with antibodies specific for gp120 peptide 44.

The cytokines associated with T-B correlations varied by treatment group, type of antibody epitope, and sign of correlation. IL-2 was the most common cytokine for correlations with linear-epitope antibodies in the IFA, peptide-2, and peptide-29 groups; but it was almost absent from this type of correlations in the PBS and control-primed groups (Figs. 3 and 4). IL-5 was the most common cytokine for correlations with CD4-blocking and crossreactive (conformational) epitopes in the control and IFA groups (Table 1). A few epitope-specific T-B correlations were identified by splenocyte proliferative responses (Table 1). Most of these coincided with correlations identified by cytokine secretion.

For the peptide-2 or peptide-29 groups, positive T-B correlations were concentrated on B cells specific for the priming peptide and nearby peptides, at the expense of correlations to other B cells. In the peptide-2 group, numerous T-B correlations involving B2, B6, and B7 were observed, while correlations involving B cells for the C-terminal epitopes of gp120 were nearly absent (Fig. 4A and Table 1). Likewise, in the peptide-29 group, many correlations involving B29 and B30 were observed, while correlations involving B cells for N- and C-terminal epitopes were reduced (Fig. 4B and Table 1). Many of the B30 correlations were distinct from B29 correlations in terms of the T-cell partner, suggesting that the B30 correlations were not due to antibodies that reacted with both of the overlapping peptides 29 and 30. Many of the negative T-B correlations in the peptide-29 group involved pairings that were present as positive correlations in the unprimed groups, e.g., T5–B7, T32–B28, and T39-B6 (Table 1).

Priming with gp120 peptides virtually eliminated T-B correlations involving CD4-blocking or crossreacting (conformational) antibodies (Table 1). In the unprimed groups, both conformational antibodies and linear-epitope antibodies were correlated with many of the same T-cell responses. For example, in the IFA group, both B7 and gp120_96ZM651.8_-crossreactive (XR) antibodies were positively correlated with T_IL-2_5, T_IL-2_22, T_IL-2_29, and T_IL-2_46. In the peptide-2 group, there were numerous positive correlations with peptide-specific antibodies but almost none with conformational antibodies. For example, in the peptide-2 group, B6 antibodies were positively correlated with T_IL-2_2, but CD4-blocking antibodies were negatively correlated with T_IL-13_2 and T_TNF-α_2. Thus, T-cell help appears to have been recruited away from the conformationally-specific B cells in the groups primed with gp120 peptides. Priming with the control peptide eliminated correlations with crossreacting antibodies but not CD4-blocking antibodies.

### Effects of Peptide-priming on T-cell Responses

**Figure 6:**
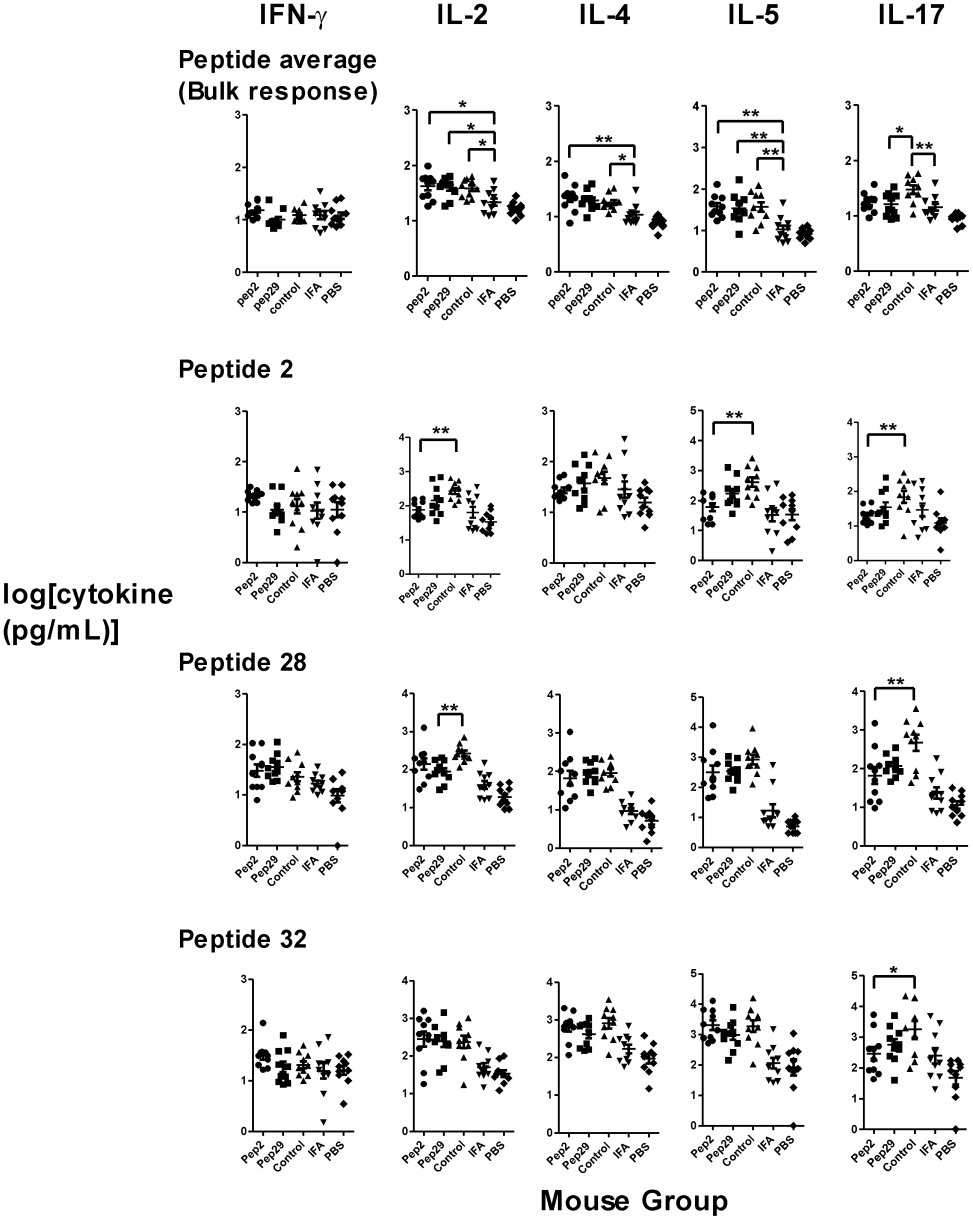
T-cell responses elicited by priming with peptide and immunization with gp120dss378. The groups of 10 mice are the same as in Figs. 3 and 4. Data are presented as mean +/− SEM for groups of ten mice. Bulk responses were compared using one-way ANOVA with Tukey’s post-test. Peptide-specific responses in gp120-peptide-primed groups were compared to those in the control-primed group using one-way ANOVA with Dunnett’s post-test. Asterisks indicate significant differences (*, p<0.05; and **, p<0.01). Bulk secretion of IL-2, IL-4, IL-5, and IL-6 (not shown) was enhanced by priming with a gp120 peptide. Bulk secretion of IL-17 was enhanced by priming with the control peptide. In contrast to the bulk response, peptide-2-specific IL-2 and IL-5 was reduced by peptide-2 priming. Likewise, peptide-28-specific IL-2 was reduced by peptide-29 priming.

Peptide priming generally enhanced T-cell responses against gp120 epitopes. The bulk T-cell response in a single mouse was defined as the average log[cytokine] secreted in response to the 38 peptides. For all three groups of peptide-primed mice, bulk IL-2, IL-4, IL-5, and IL-6 responses were significantly elevated or trending upward in comparison to responses for mice given IFA without peptide (Fig. 6 and data not shown). The bulk IL-17 response was elevated for the control-primed group but not for the groups primed with gp120 peptides. Bulk IL-10, IL-13, IFN-γ, and TNF-α were not significantly affected by peptide priming.

For individual peptide-specific T-cell responses, priming induced the largest increases for the peptides that stimulated the largest responses in the unprimed mice (Fig. S1). However, certain responses associated with the priming peptide were reduced in the primed animal groups, as compared to the same responses in the control-primed animals. In particular, the IL-2 and IL-5 responses to peptide 2 were reduced for the peptide-2 group, but the responses to all other individual peptides were indistinguishable from those in the control group (unaffected responses represented by peptides 28 and 32). Likewise, the IL-2 response to peptide 28 was reduced in the peptide-29 group, but the responses to other peptides were indistinguishable from those in the control group (Fig. 6, unaffected responses represented by peptides 2 and 32). Individual IL-17 responses generally tracked with the bulk IL-17 response, which was elevated in the control-primed group, compared to the groups primed with peptide 2 or peptide 29.

### Effects of Peptide-priming on Antibody Responses

**Figure 7:**
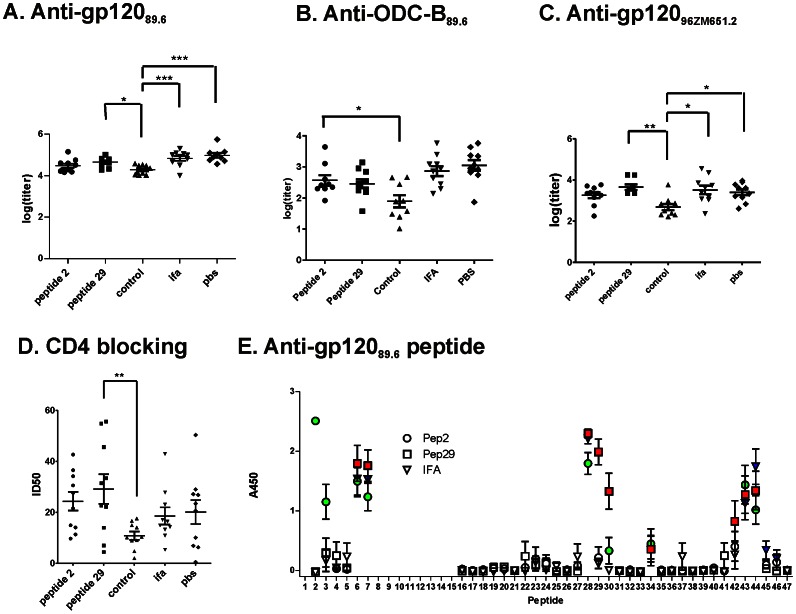
Peptide priming affects antibody responses to gp120dss378. The groups of 10 mice are the same as in Figs. 3 and 4. A-C, Log(titer) for serum IgG reaction with the indicated antigen. D, Serum dilution that obtained 50% inhibition of CD4 binding to gp120_89.6_ (ID_50_). E, reaction of IgG with gp120 peptides of serum at a dilution of 1∶100. Data are presented as mean +/− SEM for the same groups of ten mice as presented in Figs. 3 and 4. Data for peptides in which the serum from at least three mice in the group reacted with A450>0.2 are highlighted in color. Asterisks indicate significant differences (*, p<0.05; **, p<0.01; and ***, p<0.001). Data for peptide-2, peptide-29, and control-primed groups are representative of two identical experiments. Antibody reactions with protein antigens were reduced in the control-primed groups. The average reactions with peptide 2 and 3 and with peptide 29 and 30 were increased in the peptide-2 and peptide-29 primed mouse groups, respectively.

The gp120-specific antibody titers were determined by ELISA for the reaction with recombinant gp120 from HIV_89.6_ (gp120) and the outer-domain core (ODC-B), which lacks the inner domain, V3, V4, and a segment that forms part of the bridging sheet. In addition, titers were determined for the crossreaction with gp120_96ZM651.8_ and CD4 blocking. The gp120 titer was not affected by the pre-vaccination treatment with IFA or priming with a gp120 peptide, but it was reduced by approximately one half log by priming with the control peptide (Fig. 7A). The basis for the reduction in antibody is unclear but may involve a diversion of immune resources toward the control peptide. Although there was no significant T-cell or antibody reaction to the control peptide on day 0 (Fig. S2), robust control-specific IgG reactions were observed for control-primed animals after three administrations of gp120dss378 (Fig. S3).

Antibody titers for crossreaction and CD4 blocking were similar in the treatment groups. The only significant difference in reactivity between groups was the reduced antibody titer in the control-primed animals (Figs. 7B-D). However, different antibody specificities appear to be responsible for CD4 blocking in the IFA and peptide-29 groups. In the IFA group, CD4 blocking correlated with gp120_96ZM651.8_ crossreactivity (r^2^ = 0.52, p  = 0.02) but not with gp120 reactivity (r^2^ = 0.10). In contrast, in the peptide-29 group, CD4 blocking correlated with gp120 reactivity (r^2^ = 0.63, p  = 0.01) but not with gp120_96ZM651.8_ crossreactivity (r^2^ = 0.01). In the peptide-2 and control-primed groups, CD4 blocking was not correlated with any other antibody reactivity. In the PBS group, CD4 blocking was correlated with gp120_96ZM651.8_ crossreactivity (r^2^ = 0.52, p  = 0.03) and almost as well with gp120 reactivity (r^2^ = 0.48, p  = 0.06).

In the absence of priming, peptide-specific antibodies were directed mainly to peptides from the amino-terminal region, V3 loop, and V5 loop (Fig. 7E). In sera from peptide-2 animals, reactivity for peptides 2 and 3 was elevated. In sera from peptide-29 animals, reactivity for peptides 29 and 30 was elevated.

## Discussion

Large but typical mouse-to-mouse variability in peptide-specific T-cell and antibody responses enabled the identification of T-B correlations that are likely to indicate antigen-specific T-cell help to B cells. We posit that this variability in the response is not due to experimental parameters, such as the amount of immunogen administered or preexisting conditions in the individual mice. Variation in experimental parameters cannot explain peaks and gaps in the profiles of T-cell response that do not follow the overall immune response. For example, in the IFA-treated mice #1, #2, and #7, the IL-2 response to peptides 17, 24, and 30 ranged over an order of magnitude (Fig. S4A), and a different mouse responded most strongly to each peptide. Even greater mouse-to-mouse variability is present in the epitope-specificity of antibody responses (Fig. S4B). As for the T-cell responses, the presence or absence of a particular specificity of antibody does not track with the overall antibody response, suggesting that a stochastic process determines which epitopes are represented in a particular mouse. Stochastic molecular mechanisms that regulate cell fate have been suggested to be a major source of variability in immune responses [27]. Such mechanisms can yield variability in individual cell fates while maintaining stable overall populations of cell types.

Positive correlations of gp120_96zm651.8_-crossreactive and CD4-blocking antibody titers with bulk IL-2 and IL-5 most likely indicate antigen-specific T-cell help to B cells. The bulk cytokine output of T cells stimulated by a broad sample of gp120 peptides provides a good representation of the overall available T-cell helper function. Correlations involving IL-2 and IL-5 were observed in both the control-primed and IFA-treated mouse groups, which were the most similarly gp120-immunized pair of groups. Both of these cytokines support antibody production. IL-2 serves as a growth factor for T-cell proliferation. The classic Th2 cytokine IL-5 promotes B-cell maturation and is associated with production of immunoglobulins of multiple isotypes [28], [29]. Bulk levels of the other classic Th2 cytokines, IL-4 and IL-13, were not correlated with overall antibody titers, although epitope-specific T_IL-4_-B and T_IL-13_-B correlations were observed. The Th2 cytokines are thought to promote defense against parasites and contribute to atopic disorders. Remarkably, in one of the few previous studies reporting T-B correlations, the correlated responses were specific for peanut allergens in peanut-allergic subjects [18]. No correlation was observed for tetanus-specific responses in the same subjects. These observations raise the possibility that T-B collaboration, mediated at least in part by IL-5, promotes the development of high-affinity conformational antibodies, which are typical in allergy.

The enhancement of bulk IL-2, IL-4, and IL-5 T-cell responses in the gp120-peptide primed groups could provide part of the mechanism that reshapes T-B correlations. B cells will efficiently take up boosted gp120 protein and present it to gp120-primed T cells in the germinal centers (GC). Thus, protein-primed T cells can be induced to secrete IL-2, IL-4, and IL-5 by interactions with the peptide-primed B cells. Although Tfh cells are most important for GC development, they do not produce high levels of the correlated cytokines [30]. As yet, the detailed roles played by Tfh and other T-cell types in immunoglobulin class switching, antibody production, and affinity maturation remain muddled. Issues are especially complicated by the apparent ability of T cells to transform back and forth between some phenotypes, such as between Tfh-like and Th2-like [30].

T-cell responses specific for the primed gp120 peptides were not elevated in the manner observed for the bulk T-cell responses. On the basis of the study by Recher and coworkers with lymphocytic choriomeningitis virus [31], priming with an envelope peptide in IFA was expected to reduce the IFN-γ responses to that peptide following exposure to the protein. Effects on IFN-γ responses were not observed in the present study, although lower IL-2 and IL-5 were found for peptide 2 in the peptide-2 group, and lower IL-2 was found for peptide 28 in the peptide-29 group, relative to the control-primed group (Fig. 6). The circumstances of protein immunization may account for the effect on IL-2 and IL-5. Here, the protein immunization employed the mixed Th1/Th2 adjuvant, a mutant heat-labile toxin. In the Recher study, the protein immunization was mediated by a viral infection that strongly skews the immune response toward Th1. The lower IL-2 and IL-5 responses to gp120-primed peptides may in fact represent no change relative to the unprimed responses. The increased peptide-average (bulk) IL-2 and IL-5 discussed above is dominated by T cells newly primed by the gp120 protein; whereas the peptide-primed T cells may be beyond a developmental stage that can be affected by the protein boost.

In the PBS-treated group, correlations of bulk IL-6 and bulk TNF-α with different antibody subsets suggest the operation of complex mechanisms of intercellular interaction. Since these cytokines are not directly associated with T-cell help to B cells, these observations will guide future studies that address mechanisms in greater detail. The correlation of bulk IL-6 with gp120-reactivity could be due to the role of IL-6 in the development of follicular helper T cells [32]. The correlation of bulk TNF-α with CD4-blocking antibodies in the PBS-treated group could be due to suppression of CD4+ T-cell proliferation by accessory cells responding to this cytokine [33]. Reduced CD4+ T-cell proliferation was associated with increased neutralizing antibody titers in mice infected with lymphocytic choriomeningitis virus [31]. We speculate that reduced CD4+ T-cell proliferation enhances competition among B cells for T-cell help. Enhanced competition could lead to preferred T-B collaborations that promote affinity maturation in the collaborating B cells. The fact that the PBS-treated mice exhibited the largest number of negative epitope-specific T-B correlations is consistent with a higher level of competition between B cells for T-cell help in this mouse group.

The control-primed group exhibited larger IL-17 responses to gp120 peptides, and bulk IL-17 correlated with gp120-specific antibody. These results suggest that priming with the control peptide affected the phenotype of gp120-specific T cells that were primed later by the protein boost. IL-17 promotes B-cell development and germinal center formation, and the frequency of Th17 cells has been correlated with B-cell levels in B-cell deficient as well as healthy human subjects [10], [34], [35]. At least one of the gp120-specific, IL-17-producing T-cell lines was recruited to help the control-specific B cells, as suggested by the correlation of control-specific antibodies with T cells specific for peptide 40. The T_IL-17_40-B_control_ correlation could have been due to bystander effects [36], [37], [38], or the B_control_ cells may have acquired gp120 by a non-specific uptake mechanism and then presented the cognate epitope.

A handful of studies undertaken in vitro have shown that the strength of response by specific T cells depended on the specificity of the presenting B cells, the phenomenon of T-B collaboration [21], [22], [39], [40], [41], [42]. However, T-B collaboration has rarely been invoked for studies in vivo. In the present study, a B-cell directed bias toward development of certain T-B collaborations could account for both positive and negative correlations. Positive correlations arise from the well-established dependence of B-cell development on T-cell help. Negative correlations arise from competition of B-cell subsets for antigen or T-cell help – the losing B cells negatively correlate with T cells that help the winning B cells.

The mechanism for epitope-specific T-B collaboration has been proposed to derive from effects of the BCR (or antibody) on antigen processing and presentation [22]. Particular B cell subsets would favor presentation to particular T-cell subsets, and the B cells would in turn receive more T-cell help. A direct “hand-over” of antigen fragments from the BCR to MHC II proteins has been proposed [43]. The primed B cells could also provide domain-sized fragments of the antigen to other APCs [44], [45].

We are unable to rationalize the observed patterns of T-B collaborations using the existing theory of T-B collaboration, wherein an antibody protects or exposes a singular T-cell epitope that eventually stimulates the cognate helper T-cell. Most dominant B-cell epitopes correlated with multiple T-cell epitopes from disparate locations in gp120. Although the potential consequences of antibody binding to intact gp120 are complex, we cannot envision mechanisms of processing or presentation that could produce the observed T-B collaboration patterns. We submit that the formation of T-B collaborations is not as deterministic as suggested by the conventional theory of T-B collaboration. T-B collaborations could depend to a large extent on the history of T-B interactions in the individual, starting with stochastic encounters between newly activated T cells and B cells. In the present study, priming-induced shifts in epitope-specific T-B correlations suggest that preexisting B cells can shape the development of T-B collaborations.

Priming with peptide 29 gives B29 cells a head-start in the recruitment of T cells. At the same time, other B cells are competing with B29 cells for antigen and/or T-cell help. For example, B29 was correlated with T_IL-2_17 and T_IL-2_40; whereas, B28 was *anti*-correlated with T_IL-2_17 and T_IL-2_40. For this pair of overlapping B-cell specificities, binding of the antibody and BCR to the respective epitopes could be exclusive, and B29 antibody could block recognition and uptake of antigen by B28 cells. The more help B29 cells get from T_IL-2_17 and T_IL-2_40, the more effectively they block antigen uptake by B28 cells. Alternatively, the B cells may compete for T-cell help. In this scenario, the more help B29 cells get from T_IL-2_17 and T_IL-2_40, the more effectively they sequester T-cell help from B28 cells. B-cell competition for T cells has been attributed to the ability of B cells to trap T cells in T-B border clusters prior to germinal center formation [46].

In peptide-29-primed mice, the B30 antibody was correlated with T_IL-4_5, and the B6 antibody was *anti*-correlated with T_IL-4_5. One could envision an inverted form of the classic T-B collaboration, wherein B6 antibody somehow inhibits presentation to T_IL-4_5; but this possibility appears to be excluded because B6 was positively correlated with T_IL-2_5 in peptide-2-primed mice. The more likely possibilities are as described above for B28 and B29. B30 and B6 cells compete for limited antigen, and B30 cells dominate the competition in proportion to help available from T_IL-4_5. Alternatively, B30 cells sequester T_IL-4_5 cells in a compartment that is unavailable to B6 cells; and the effectiveness of T_IL-4_5 sequestration is proportional to help from T_IL-4_5. The latter scenario at first seems paradoxical because increased T_IL-4_5 coincides with reduced availability of T_IL-4_5 to B6 cells. The paradox could be resolved if the availability of T_IL-4_5 cells is limited and if enhanced B30-T_IL-4_5 collaboration dramatically improves T_IL-4_5 sequestration.

The following model can explain several features of T-B correlations observed in this work. Peptide priming activates a B-cell subset that is specific for the peptide. This is evident by the development of IgG and/or IgM specific for the priming peptide in mice that were primed but not boosted with gp120dss378 (Fig. S2). The protein boosts the B cell response for the primed peptide by providing a source of the peptide and a spectrum of T-helper cells. As the response to the protein develops, the primed B-cell subsets dominate because they receive help from the dominant T-cells and reduce the availability of T cells to other B-cell subsets. We think it plausible that the degree to which B cells sequester T cells could be a function of the size and/or number of T-B border clusters formed prior to germinal center formation.

In the absence of peptide priming, B-cell dominance and T-B collaboration is not channeled toward a particular B-cell subset. The abundance of antigen presentation by different B-cell subsets has been shown to determine which will receive T-cell help [47]. If the various naïve B cells occur at comparable frequencies, then competition for recruitment of T cells may depend acutely on both BCR affinity for the antigen and the circumstances of immunization. Among the five mouse groups studied here, the IFA and control groups shared the most T-B correlations. Thus, the most similar circumstances of immunization yielded the most similar T-B collaborations.

Priming with gp120 peptides eliminated T-B correlations involving conformational antibodies by recruiting T cells to collaborate with peptide-specific B cells. If the T-B correlations in the IFA group are taken as the “unperturbed” pattern, then priming with peptide-2 and peptide-29 reassigned almost all collaborations to nearby linear epitopes. In contrast, priming with the control peptide replaced only a fraction of correlations involving conformational epitopes, namely those for crossreactive epitopes. Thus, control-primed B cells were much less effective in the recruitment of gp120-specific T cells, as expected from their reduced ability to take up and present gp120. This is consistent with the pattern of linear-epitope correlations in the control-primed group being similar to that for the IFA-treated group. We speculate that the collaborations involving crossreactive epitopes were selectively replaced by control priming because the crossreactive B cells have the least affinity for the antigen prior to affinity maturation. The loss of collaborations involving conformational antibody in the peptide-primed groups suggests that immunization strategies targeting conformational HIV-neutralizing epitopes should avoid exposure of unfolded HIV envelope protein in the prime.

The reshaping of T-B collaborations observed in this study suggests that antibody-epitope priming could be used to improve immunization against HIV. The peptides used for priming in this study were chosen to facilitate the analysis of the response. As expected from their small contribution to the mass of gp120, priming with these epitopes had a negligible effect on the overall gp120 antibody response. The gp120-specific IgG titers in the gp120-peptide primed and unprimed groups were indistinguishable, and the breadth of peptide specificity of the antibodies were similar (excluding reactivity with primed peptides). Crossreactive and CD4-blocking antibody levels also were indistinguishable in the gp120-peptide primed and unprimed groups. Thus, the overall level of gp120-specific antibody was insensitive to the shifts in T-B collaboration induced here. Nevertheless, protein boosting greatly enhanced the antibody response to the priming peptides. In addition, the CD4-blocking activity correlated with different antibody subsets (crossreactive vs. gp120-specific) in the IFA-treated and peptide-29 mouse groups, suggesting that CD4 blocking is mediated by antibody directed against different epitopes. Thus, priming with an immunogen that displays an HIV-neutralizing epitope should be able to enhance T-B collaborations that support affinity maturation of HIV-neutralizing antibodies.

## Supporting Information

Figure S1 **Profiles of cytokine secretion log[cytokine(pg/ml)] in response to individual peptides.** Data are presented as mean +/− SEM for groups of ten mice. Individual mouse responses are available in Table S2.
(PDF)

Figure S2 **Evidence of immune priming was examined in a preliminary study of mice that were treated with only peptide 2, peptide 29, or control peptide.** The peptides emulsified in incomplete Freund’s adjuvant (IFA) were administered to groups of four BALB/c mice in the peritoneal cavity over a period of six days (day -9 to day -3, following the scheme in A), as described by Lang et al. (2007). On day 0, the animals were sacrificed; and cellular and humoral responses were examined. When splenocytes of each group were tested for IL-2 ELISpot formation (two animals) or proliferation (two animals), only the peptide-2-primed animals responded (B). Since peptide 29-specific T cell responses were previously observed following immunization with gp120, the failure of peptide 29 to prime T cells may be due to any of several distinct features of the immunization, such as immunogen, route, or adjuvant. Serum IgM and IgG from peptide-2-primed mice reacted with peptide 2, but only the IgM from peptide-29-primed mice reacted with peptide 29 (C and D). The lack of IgG reactivity for peptide 29 is consistent with the absence of a T-cell response to this peptide. Priming with the control peptide elicited neither IgG nor IgM that reacted with the control peptide. Data are presented as mean +/− SEM for groups of four mice. Reference: Lang, K.S., Hegazy, A.N., Lang, P.A., Eschli, B., Lohning, M., Hengartner, H., Zinkernagel, R.M., and Recher, M. (2007). "Negative vaccination" by specific CD4 T cell tolerisation enhances virus-specific protective antibody responses. PLoS ONE 2, e1162.
(PDF)

Figure S3 **Reactivity of serum samples (diluted 1∶100) determined by ELISA on plates coated with control peptide.** Serum from the Control-primed animals reacted with the Control peptide.
(PDF)

Figure S4 **Variation in peptide-specific IL-2 responses (A) and antibody reactions (B) of individual mice in the same group (peptide-29 primed).** In both A and B, a different mouse yields the largest response (oval) to a given peptide, suggesting that the variation in responses is not related to the overall strength of immunization.
(PDF)

Table S1 **Thirty-eight peptides spanning the sequence of HIV_89.6_ gp120, except V1–V2**. Peptide sequences including cysteines as the carbamidomethyl derivatives were as described by Dai, G., Steede, N.K., and Landry, S.J. (2001), Allocation of helper T-cell epitope immunodominance according to three-dimensional structure in the human immunodeficiency virus type I envelope glycoprotein gp120, J Biol Chem *276*, 41913-41920.
(PDF)

Table S2 **Peptide-specific T-cell and B-cell responses (Excel file).**
(XLSX)
