# Supplementary figures and images for: Shaping T Cell – B Cell Collaboration in the Response to Human Immunodeficiency Virus Type 1 Envelope Glycoprotein gp120 by Peptide Priming
Source: PLoS One. 2013 Jun 11;8(6):e65748. doi: 10.1371/journal.pone.0065748 (PMC3679139; doi:10.1371/journal.pone.0065748)

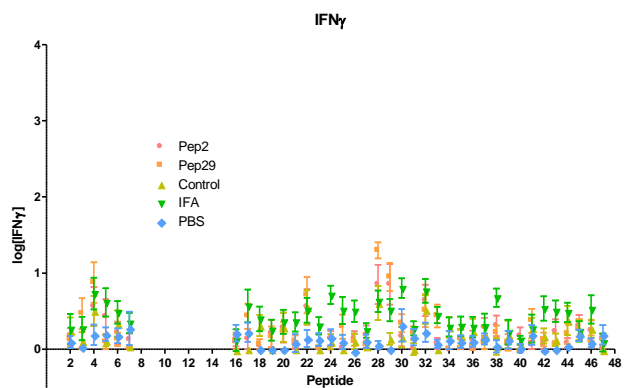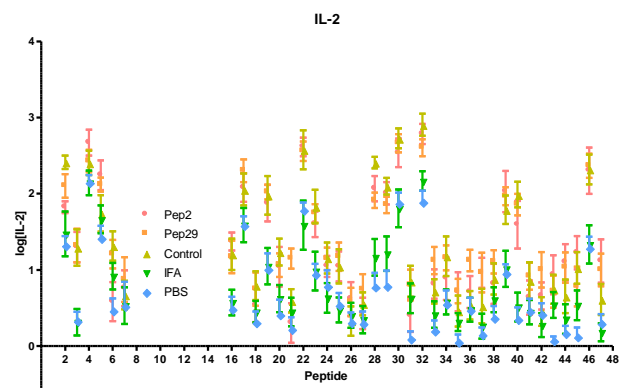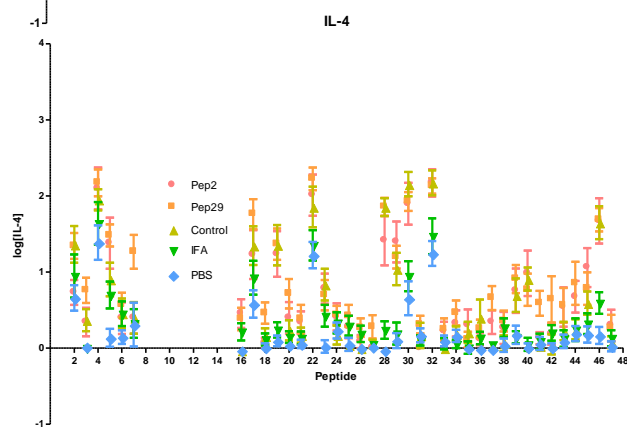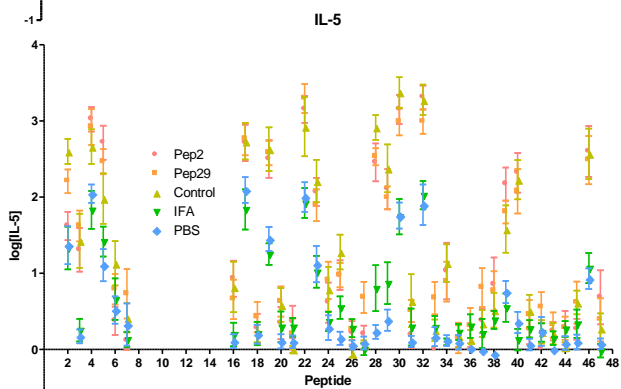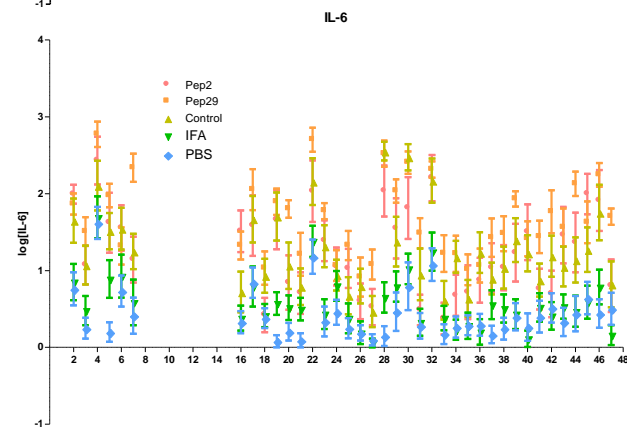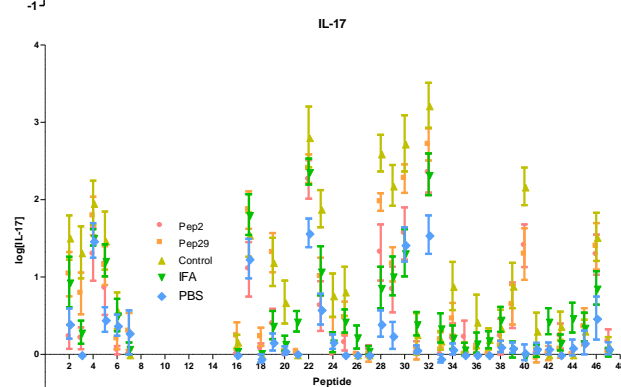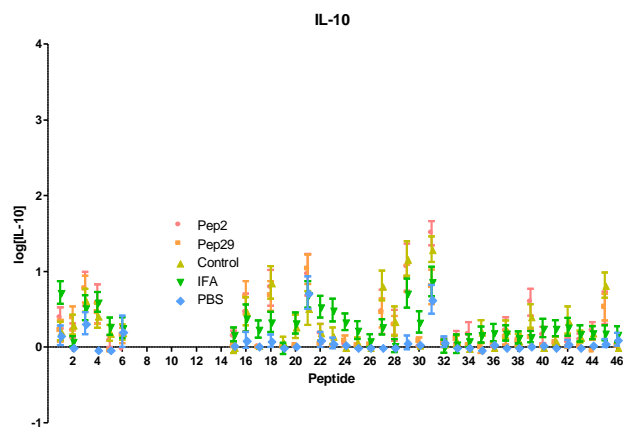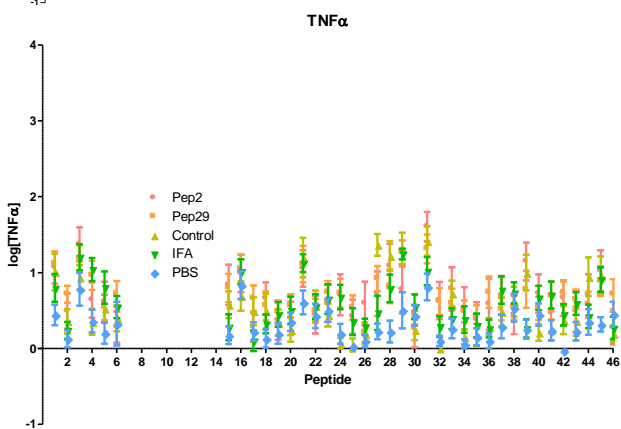

Supplement: Figure S1 — Profiles of cytokine secretion log[cytokine(pg/ml)] in response to individual peptides. Data are presented as mean +/− SEM for groups of ten mice. Individual mouse responses are available in Table S2. (PDF) [file pone.0065748.s001.pdf]

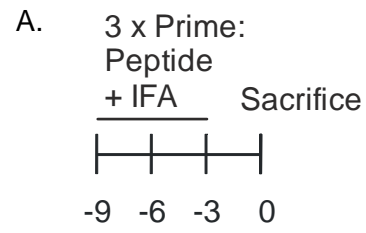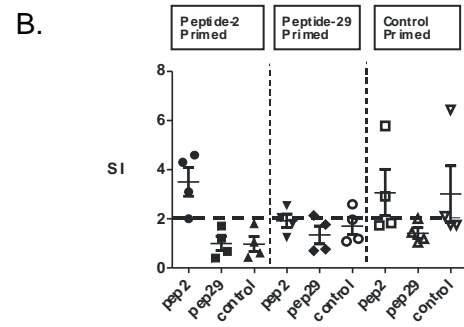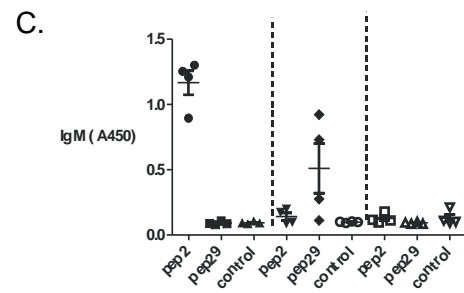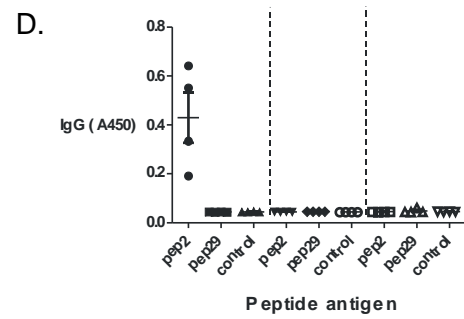

Supplement: Figure S2 — Evidence of immune priming was examined in a preliminary study of mice that were treated with only peptide 2, peptide 29, or control peptide. The peptides emulsified in incomplete Freund’s adjuvant (IFA) were administered to groups of four BALB/c mice in the peritoneal cavity over a period of six days (day -9 to day -3, following the scheme in A), as described by Lang et al. (2007). On day 0, the animals were sacrificed; and cellular and humoral responses were examined. When splenocytes of each group were tested for IL-2 ELISpot formation (two animals) or proliferation (two animals), only the peptide-2-primed animals responded (B). Since peptide 29-specific T cell responses were previously observed following immunization with gp120, the failure of peptide 29 to prime T cells may be due to any of several distinct features of the immunization, such as immunogen, route, or adjuvant. Serum IgM and IgG from peptide-2-primed mice reacted with peptide 2, but only the IgM from peptide-29-primed mice reacted with peptide 29 (C and D). The lack of IgG reactivity for peptide 29 is consistent with the absence of a T-cell response to this peptide. Priming with the control peptide elicited neither IgG nor IgM that reacted with the control peptide. Data are presented as mean +/− SEM for groups of four mice. Reference: Lang, K.S., Hegazy, A.N., Lang, P.A., Eschli, B., Lohning, M., Hengartner, H., Zinkernagel, R.M., and Recher, M. (2007). "Negative vaccination" by specific CD4 T cell tolerisation enhances virus-specific protective antibody responses. PLoS ONE 2, e1162. (PDF) [file pone.0065748.s002.pdf]

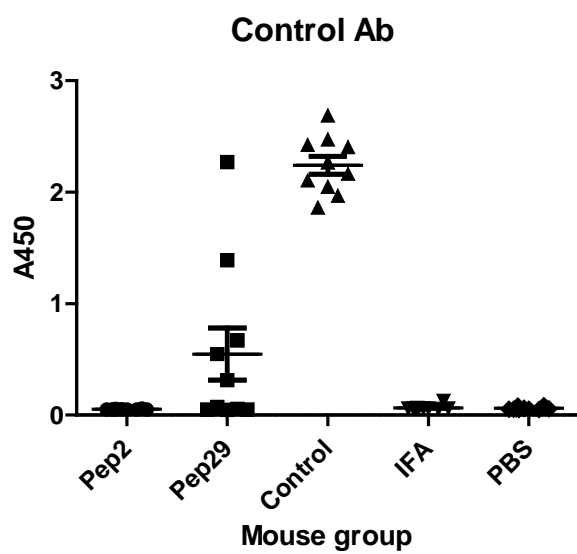

Supplement: Figure S3 — Reactivity of serum samples (diluted 1∶100) determined by ELISA on plates coated with control peptide. Serum from the Control-primed animals reacted with the Control peptide. (PDF) [file pone.0065748.s003.pdf]

A. T-cell Response

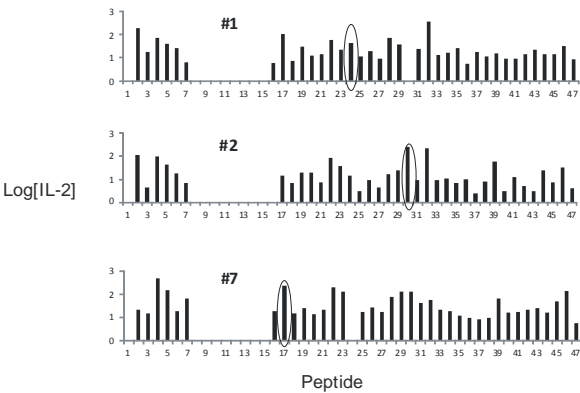

B. IgG Response

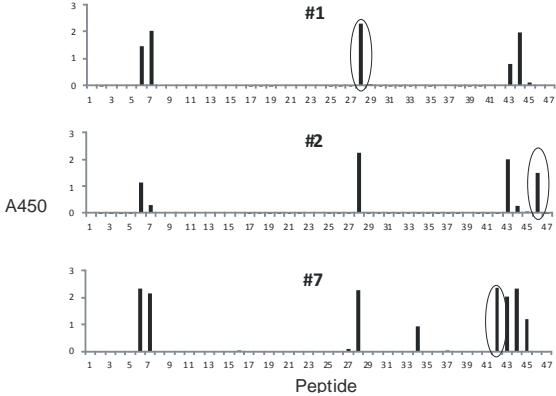

Supplement: Figure S4 — Variation in peptide-specific IL-2 responses (A) and antibody reactions (B) of individual mice in the same group (peptide-29 primed). In both A and B, a different mouse yields the largest response (oval) to a given peptide, suggesting that the variation in responses is not related to the overall strength of immunization. (PDF) [file pone.0065748.s004.pdf]
