# Supplementary material for: Shaping T Cell – B Cell Collaboration in the Response to Human Immunodeficiency Virus Type 1 Envelope Glycoprotein gp120 by Peptide Priming
Source: PLoS One. 2013 Jun 11;8(6):e65748. doi: 10.1371/journal.pone.0065748 (PMC3679139; doi:10.1371/journal.pone.0065748)
Supplement: Table S1 — Thirty-eight peptides spanning the sequence of HIV89.6 gp120, except V1–V2. Peptide sequences including cysteines as the carbamidomethyl derivatives were as described by Dai, G., Steede, N.K., and Landry, S.J. (2001), Allocation of helper T-cell epitope immunodominance according to three-dimensional structure in the human immunodeficiency virus type I envelope glycoprotein gp120, J Biol Chem 276, 41913-41920. (PDF) [file pone.0065748.s005.pdf]

Peptide	Sequence Number		Sequence
2	40	59	GVPVWREATTTLFCASDAKA
3	50	69	TLFCASDAKAYDTEVHNVWA
4	60	79	YDTEVHNVWATHACVPTDPN
5	70	89	THACVPTDPNPQEVVLGNVTE
6	80	99	PQEVVLGNVTENFNMWKNNM
7	90	109	ENFNMWKNNMVDQMHEDIIS
16	180	199	LDVVPIENTNNTKYRLISCN
17	190	209	NTKYRLISCNTSVITQACPK
18	200	219	TSVITQACPKVSFQPIPIHY
19	210	229	VSFQPIPIHYCVPAGFAMLK
20	220	239	CVPAGFAMLKCNNKTFNGSG
21	230	248	CNNKTFNGSGPCTNVSTVQ
22	240	259	PCTNVSTVQCTHGIRPVVST
23	250	269	THGIRPVVSTQLLLNGSLAE
24	260	279	QLLLNGSLAEEDIVIRSENF

Peptide	Sequence Number		Sequence
25	270	289	EDIVIRSENFTDNAKTIIVQ
26	280	299	TDNAKTIIVQLNESVVINCT
27	290	309	LNESVVINCTRPNNNTRRRL
28	300	319	RPNNNTRRRLSIGPGRAFYA
29	310	329	SIGPGRAFYARRNIIGDIRQ
30	320	339	RRNIIGDIRQAHCNISRAKW
31	330	349	AHCNISRAKWNNTLQQIVIK
32	340	359	NNTLQQIVIKLREKFRNKTI
33	350	368	LREKFRNKTIAFNQSSGGD
34	360	379	AFNQSSGGDPEIVMHSFNCG
35	370	389	EIVMHSFNCGGEFFYCNTAQ
36	380	399	GEFFYCNTAQLFNSTWNVTG
37	390	409	LFNSTWNVTGGTNGTEGNDI
38	400	419	GTNGTEGNDIITLQCRIKQI
39	410	429	ITLQCRIKQIINMWQKVGKA
40	420	439	INMWQKVGKAMYAPPITGQI
41	430	449	MYAPPITGQIRCSSNITGLL
42	440	459	RCSSNITGLLLTRDGGNSTE
43	450	469	LTRDGGNSTETETEIFRPGG
44	460	479	TETEIFRPGGGDMRDNWRSE
45	470	489	GDMRDNWRSELYKYKVVRIE
46	480	499	LYKYKVVRIEPIGVAPTRAK
47	490	508	PIGVAPTRAKRRTVQREKR
